# Zinc supplementation of lactating dairy cows: effects on chemical-nutritional quality and volatile profile of Caciocavallo cheese

**DOI:** 10.5713/ajas.19.0155

**Published:** 2019-08-03

**Authors:** Andrea Ianni, Camillo Martino, Denise Innosa, Francesca Bennato, Lisa Grotta, Giuseppe Martino

**Affiliations:** 1 Faculty of BioScience and Technology for Food, Agriculture and Environment, University of Teramo, Via Renato Balzarini 1, 64100 Teramo, Italy; 2 Department of Veterinary Medicine, University of Perugia, Via S. Costanzo 4, 06126 Perugia, Italy

**Keywords:** Zinc Oxide, Friesian Cow, Caciocavallo Cheese, Lipid Profile, Lipid Peroxidation, Volatile Compound

## Abstract

**Objective:**

The aim of the present study was to investigate the effect of dietary zinc supplementation of Friesian cows on chemical-nutritional and aromatic properties of Caciocavallo cheese after 7 days (C_7_) and 120 days (C_120_) of ripening.

**Methods:**

Twenty eight Friesian cows, balanced for parity, milk production and days in milk, were randomly assigned to 2 groups. The control group (CG) was fed with a conventional complete diet, while the experimental group (zinc group, ZG) received a daily zinc supplementation of 60 mg for kg of dry complete feed. During the experimental period, the milk yield was monitored and samples of milk and caciocavallo cheese were collected and analyzed for chemical-nutritional composition and aromatic profile.

**Results:**

The enrichment of dairy cows diet with zinc, did not influence milk yield and composition, however a marked reduction of somatic cell count was evidenced. Both in milk and cheese the ZG samples were characterized by a lower concentration of satured fatty acids and an increase in oleic, vaccenic and rumenic acids. The aromatic profile of dairy products was also positively affected by dietary zinc intake, with an increase in concentration of carboxylic acids, esters and lactones.

**Conclusion:**

The present results suggest a positive role of dietary zinc intake in improving the quality of bovine milk and related cheese, in particular for the increase in concentration of bioactive fatty acids such as rumenic acid. The changes evidenced in cheese through the analysis of the volatile profile, would be consistent with the development of interesting organoleptic properties, although further evaluations should be performed to confirm the consumer acceptability of these changes.

## INTRODUCTION

High yielding animals require feeding strategies which guarantee the right contribution of all the necessary microelements, such as zinc (Zn), manganese, copper, cobalt, iodine and selenium. The Zn is an ubiquitous element in cells and is one of the most studied minerals of the twenty-first century as it is practically involved in every function of the body. It is an essential component of several metallo-enzymes [1] and has been found as a relevant element for many transcription factors which control gene expression hence possesses a fundamental role in cell division, development and differentiation [2]. Zn also plays a relevant role in the stabilization of RNA, DNA, and ribosomes, is involved in insulin production, has antioxidant effects [3], is crucial for maintenance of integrity and the barrier function of skin and is involved in the immune system [4]. The body inability to store Zn, makes necessary a constant dietary supply in order to avoid the onset of a wide range of pathological conditions, such as skin parakeratosis, reduced or cessation of growth, general debility, lethargy and increased susceptibility to infection [5].

Both human and veterinary medicine have paid particular attention to the development of administration protocols and supplements containing mostly inorganic Zn (oxide, sulphate), in an attempt to provide a sufficient amount of this microelement in both animals and humans. Generally, Zn utilization from feed depends upon the process of digestion, absorption and metabolism which requires further investigation, although the recent advances in Zn nutrition like the identification of Zn transporters and the role of free Zn ions have led to new insights in understanding the metabolism and homeostasis of Zn [6]. In ruminants, significant Zn absorption occurs through the rumen which, is the function of source, concentration of Zn and the resident time of feed [7]. Studies on lambs indicate similar bioavailability of Zn sulfate and Zn oxide [8,9] and, although the mode of action is unclear, research suggests that feed enriched with certain organic forms of Zn can improve animal production responses if compared with those observed in ruminants that are supplemented only with inorganic Zn [10]. The apparent Zn absorption from Zn methionine and Zn oxide was similar when fed to lambs affected by Zn deficiency, although the urinary excretion of the microelement tended to be lower in lambs fed Zn methionine, suggesting an higher Zn retention [11].

Regarding the influence of dietary Zn intake on the quality of milk and dairy products, some evaluations have been made on ruminants fed with diets enriched both with organic and inorganic Zn. Salama et al [12] showed that milk yield was not significantly affected by Zn-methionine intake in dairy goats. Furthermore, the percentages of protein, lactose, fat, nonfat solid, total solid, and density of milk were not significantly different between treatments. Later, Sobhanirad et al [13] confirmed this behaviour in dairy cows, whose milk did not display variations between different Zn sources. In addition to this, it is also important to emphasize that an adequate microelements bioavailability is of fundamental importance in order to support the ruminal fermentation mediated by the enzymatic activities of ruminal microbes which directly contribute to the chemical-nutritional quality of milk and products derived from it. Ruminant products represent the only natural sources of conjugated linoleic acids (CLAs) for human diet, besides conferring significant amounts of various trans fatty acids (FAs), especially monounsaturated isomers [14].

The present study aimed to deeply investigate the role of dietary Zn supplementation in influencing the nutritional quality of milk and related dairy products obtained from lactating dairy cows, with particular attention on FAs composition, oxidative stability and aromatic profile.

## MATERIALS AND METHODS

### Experimental design, diets, and sampling

The experimental plan was performed according to Directive 2010/63/EU of the European Parliament (European Union, 2010) and Directive 86/609/EEC (European Economic Community, 1986), which deal with the protection of animals used for scientific purposes.

Twenty eight healthy Friesian cows, homogeneous for age (43.5±3 months) and lactation days (76±17 days) were used in this study. Animals were randomly divided into two groups of fourteen cows each: a control group (CG) and an experimental group (zinc group, ZG) whose diet was supplemented with ZnO. The cows were housed for the entire trial period in two separate areas of free housing with an access to an identical feeding area. The study was conducted for a period of 56 days, in which all animals received about 22 kg/head/d of dry matter of total mixed rations (TMR) whose composition, reported in Table 1, was defined taking into account the parameters reported on the seventh edition of Nutrient Requirements of Dairy Cattle [15]. Samples of TMR were analyzed, according to AOAC methods [16], for crude protein (method 930.15), ether extract (method 920.39), crude fiber (method 962.09), and ash (method 942.05); detergent procedures reported by Van Soest et al [17] were used for the determination of neutral detergent fiber and acid detergent fiber.

The CG received, in the form of “unifeed” during the entire trial, a complete food formulated taking into account the nutritional needs of cows in mid-lactation, and guaranteeing each animal the daily Zn requirement of about 40 mg for kg of dry complete feed. The ZG received the same complete food, however the daily ration of each cow was enriched with additional 60 mg Zn for kg of dry complete feed in order to obtain a total intake of about 100 mg/kg. For The rations were prepared with ZnO, and the dose management was performed according to the Regulation (EC) No. 1831/2003 of the European Parliament on additives for use in animal nutrition.

On the 56th day the milk was collected separately for each group and manipulated in the same way during cheese-making to obtain the caciocavallo cheese, according to the manufacturing protocol reported below. Bulk milk was firstly filtered, and then heated to 36°C to 38°C and enriched with thermophilic microbial lactic flora. The curd formation was obtained by the addition of calf rennet, and after the completion of the coagulation the curd was broken in order to remove the whey. The mass was left in whey for at least two hours and then moved to another container with water at 80°C, to facilitate the spinning. Lastly, the cheeses were shaped by hand, tied at the top and moved to another container, with cold water, for hardening. Then they were left to soak in brine for 12 to 20 hours. After salting, cheeses were left to ripen at 10°C to 15°C and a relative humidity of 85% to 90%. In order to evaluate changes in the chemical composition and quality attributes due to ripening, sampling and analyses on caciocavallo cheese were carried out after 7 days (C_7_) and 120 days (C_120_) from the cheese-making. Samples, collected in triplicate from three different cheese-makings, were partly immediately analyzed and partly packed under vacuum and frozen at −20°C until analysis.

### Chemical analysis of milk and caciocavallo cheese

Chemical composition of milk (fat, protein, casein, lactose, and urea) was determined by MilkoScan FT 6000 (Foss Integrator IMT; Foss, Hillerød, Denmark), while somatic cells count (SCC) and total bacterial count (TBC) were performed using respectively the Fossomatic TM FC and the BactoScan FC (Foss, Hillerød, Denmark). In cheese, the evaluation of pH, dry matter, total proteins, lipids, and ash were performed as previously described by Tofalo et al [18].

For the determination of total amount of Zn in milk and cheese, samples were firstly mineralized by dry incineration, and then subjected to atomic absorption spectrophotometry using an air/acetylene flame [19]. The determination of Zn was performed by referring to a calibration and results were expressed in mg/kg.

Milk lipid fraction was extracted according to the AOAC official method [20], while in cheese, the extraction was performed as described by Domagala et al. by using a mix of chloroform and methanol (2:1, v/v) [21]. Trans-methylation of lipid extracts and separation of fatty acyl methyl esters was performed following the procedure reported by Ianni et al [22]. Peak areas were quantified using ChromeCard software, and the relative value of each individual FA was expressed as a percentage of the total FA. The value of each FA was used to calculate the sum of saturated fatty acids (SFA), monounsaturated fatty acids (MUFA), and polyunsaturated fatty acids (PUFA). Considering the values associated with each FA, atherogenic and thrombogenic indices (AI and TI, respectively) were calculated in milk and cheese using the formulas proposed by Ulbricht and Southgate [23]; desaturation index (DI) was calculated as proposed by Mele et al [24].

### Evaluation of the oxidative stability in cheese by thiobarbituric acid reactive substances-test

Fat oxidation was evaluated by measuring thiobarbituric acid reactive substances (TBARS). The analysis was performed according to the procedure reported by Grotta et al [25] with slight modifications. For each sample, an aliquot of 4 g of frozen cheese was mixed, within 2 min of sample withdrawal from the freezer, with 400 μL of 0.1% of butylated hydroxytoluene in methanol to stop the oxidation process. The mixture was homogenized with Ultra Turrax T-25 high speed homogenizer (IKA, Staufen, Germany) in 40 mL of an acqueous solution of 7% trichloroacetic acid, and then subjected to distillation. An aliquot of 2 mL of each distillate was mixed with an equal volume of a 0.02 M thiobarbituric acid solution in 90% acetic acid. The solution was kept for one hour in a thermostated bath at 80°C, and only after cooling, the absorbance at 534 nm was evaluated with a spectrophotometer (JENWAY 6305 UV/vis, Jenway, Essex, UK). The amount of malondialdehyde (MDA) of each sample was calculated by using a calibration curve and results were expressed in μg of MDA per g of cheese.

### Analysis of volatile compounds

Extraction of volatile compounds (VOC) from milk and cheese samples was performed by solid-phase microextraction (SPME), and gas chromatography-mass spectrometry analysis was performed with a gas chromatograph (Clarus 580; Perkin Elmer, Waltham, MA, USA) coupled with a mass spectrometer (SQ8S; Perkin Elmer, USA) [26]. The gas chromatograph was equipped with an Elite-5MS column (length× internal diameter: 30×0.25 mm; film thickness: 0.25 μm; Perkin Elmer, USA). 5.5 g of cheese previously grated were mixed with 10 mL of saturated NaCl solution (360 g/L), and then 10 μL of internal standard solution (4-methyl-2-heptanone; 10 mg/kg in ethanol) were added. Vials were sealed with a polytetrafluoroethylene-silicone septum (Supelco, Bellefonte, PA, USA) and stirred at 55°C; VOCs were extracted from the headspace with a divinylbenzene-carboxen-polydimethylsiloxane SPME fiber (length: 1 cm; film thickness: 50/30 μm; Supelco, USA) with an exposition time of 60 min. The extracted VOCs were thermally desorbed into the gas chromatograph injector splitless mode for 1 min at 250°C. The oven temperature was held at 50°C for 1 min, increased at a rate of 3°C/min up to 200°C and held for 1 min, and then increased from 200°C to 250°C at 15°C/min and held for 15 min. Helium was used as carrier gas at a flow rate of 1 mL/min. The mass spectrometer operated in electronic impact ionization mode at 70 eV, and data were collected in full scan mode, with a scan time of 0.2 s over a mass range of 35 to 350. Source and interface temperature were held at 250°C. The VOCs were identified by comparison with mass spectra of a library database (NIST Mass Spectral library, Search Program version 2.0, National Institute of Standards and Technology, U.S. Department of Commerce, Gaithersburg, MD, USA) and by comparing the eluting order with Kovats indices.

### Statistical analysis

Statistical data processing was performed by using the SAS software, version 9.2 (2000). The data concerning the VOCs of cheese were elaborated with two-way analysis of variance, considering diet and ripening time as fixed points. Separation of means was assessed by Student’s t-test, and differences were considered significant for p<0.05.

## RESULTS

### Chemical-nutritional composition of milk and cheese

With regard to the daily milk production, no significant differences were evidenced both with reference to diet and diet-period interaction for the duration of the trial. Taking into account the chemical composition of milk (Table 2), all the analyzed parameters showed no variations. Similarly no significant differences were observed for the ureic content, TBC and pH, whereas the ZG samples showed a lower SCC with respect to the CG (p<0.05). As regards the amount of Zn, significant higher average values were detected in the ZG (3.59 vs 4.22 mg/kg in CG and ZG respectively; p<0.05).

In the case of caciocavallo cheese, no significant differences (p>0.05) in composition were induced by the feeding strategy (Table 3). Regarding the ripening time, in C_120_ samples the dry matter was significantly higher (67.70% vs 55.11% in CG and 66.21% vs 53.21 in ZG samples; p<0.01). The ripening time did not influence protein, lipids and ash whereas, as expected, an increase of the soluble nitrogen was observed in C120 samples (0.12% vs 0.073% in CG and 0.15% vs 0.077% in ZG; p<0.05) without significant variations between CG and ZG. The experimental feeding strategy positively affected the Zn amount which maintained significant higher values both in C_7_ and in C_120_ samples (p<0.05); for this parameter non variations were instead induced by ripening time.

### Fatty acid profile and lipid oxidation

The FA profile in milk and corresponding dairy products is shown in Table 4. Samples of bulk milk, obtained from ZG, evidenced an increase in the content of oleic acid (C18:1 *cis*9; p<0.05), vaccenic acid (C18:1 *trans*11; p<0.01), linoleic acid (C18:2, *n*-6; p<0.01), rumenic acid (CLA; p<0.01) ant total MUFA and PUFA (p<0.05 and p<0.01, respectively). Similarly, the evaluation of the acidic profile in caciocavallo cheese evidenced variations already evident in milk, with an increase in concentration, in the experimental group, of oleic acid (C18:1 *cis*9; p<0.01), vaccenic acid (C18:1 *trans*11; p<0.01), linoleic acid (C18:2, *n*-6; p<0.05), rumenic acid (CLA; p<0.05) ant total MUFA and PUFA (p<0.01 and p<0.05, respectively). Taking into account the obtained FA profile, calculations of AI, TI, and DI were performed, highlighting a general improvement of the health functionality of milk and cheese. The AI and TI lowered in ZG (p<0.05), whereas an increase in DI was observed (p<0.05).

**Figure 1:**
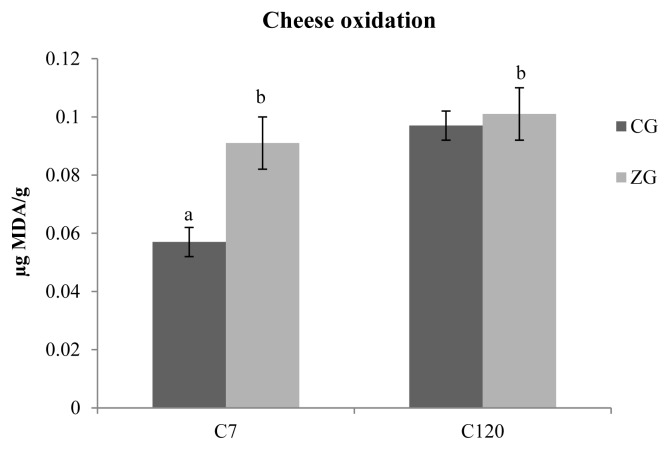
Oxidative stability of caciocavallo cheese samples obtained from the control group (CG) and the zinc group (ZG). Analysis were performed on samples obtained after 7 (C_7_) and 120 days (C_120_) from the cheese-making. The levels of thiobarbituric acid reactive substances (μg of MDA/g of cheese) significantly increased in CG samples after 120 days from the cheese-making as can be seen from the dark colored bars. The ZG samples (light colored bars) manteined similar values at the beginning and at the end of ripening.

An interesting finding of the study is the oxidative state of caciocavallo cheese after 7 and 120 days of ripening. As can be seen in Figure 1, the levels of TBARS significantly increased in CG samples after 120 days from the cheese-making (0.057 vs 0.097 μg MDA/g; p<0.05), whereas the ZG samples manteined similar values at the beginning and at the end of ripening (0.091 vs 0.101 μg MDA/g). It is important to consider the fact that at the beginning of ripening, the amount of TBARS (μg of MDA/g of cheese) in the ZG was about 1.59 times higher than the control (p<0.01).

### Aromatic profile of caciocavallo cheese

**Figure 2:**
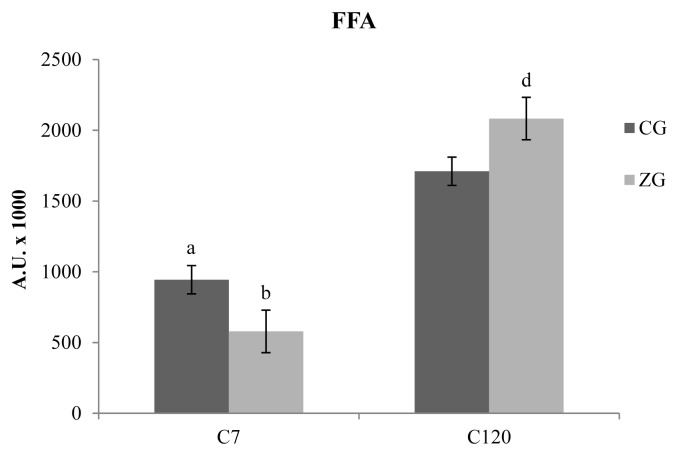
Free fatty acids (FFAs) detected in caciocavallo cheese samples obtained from control group (CG) and zinc group (ZG) after 7 (C_7_) and 120 (C_120_) days of ripening. The results obtained after 7 days of ripening, showed a higher concentrations of these compounds in CG samples (dark colored bars) with respect to ZG samples (light colored bars). One hundred and twenty days after the cheese-making, the concentration of FFAs as a whole tends to increase but can be evidenced a reversal of the phenomenon observed at C_7_, with the presence of higher concentrations of FFAs in the ZG samples with respect to the CG samples.

Several chemical families of VOCs were detected in C_7_ and C_120_ cheese samples obtained from CG and ZG. The majority of the identified compounds consists of free fatty acids (FFAs), followed by methyl ketones, methyl esters, ethyl esters, lactones, aldehydes, and alcohols, testifying the prevalence of lipolytic catabolism with respect to the proteolytic event. Regarding total FFAs (Figure 2), the results obtained after 7 days of ripening, showed a higher concentrations of these compounds in CG samples with respect to ZG samples (p<0.05). 120 days after the cheese-making, a reversal of this phenomenon was evidenced, with the presence of higher concentrations of FFAs in the ZG samples with respect to the CG samples (p<0.05). In addition to this, is also interesting to highlight the general increase of FFAs in comparison with the C_7_ samples (p<0.01); the increase is about 2 times for CG and more than three times for ZG. The analysis of individual FFAs showed differences only in C_7_ samples, with a reduction of hexanoic and heptanoic acids in the ZG (p<0.05 and p<0.01, respectively).

Concerning the other listed families of compounds (Table 5), several variations were already evident after 7 days of ripening; in particular, in ZG samples, it was possible to observe a significant increase of 2-pentanone, 2-undecanone, methyl butanoate, methyl hexanoate, nonanal and decanal, whereas, in the same samples, a reduction of ethyl butanoate, ethyl hexanoate, ethyl octanoate, δ-octalactone and hexanal was detected. Most of the variations were found after 120 days of ripening; in this case, the ZG samples were poorer in methyl ketones (2-pentanone, 2-heptanone, 2-octanone, 2-nonan-2-one, 2-nonanone; p<0.01) and richer in ethyl esters (all the ethyl esters listed in Table 5; p<0.01) and lactones (cis-γ-dodec-6-enolactone, γ-nonalactone, γ-dodecalactone, δ-decalactone, δ-dodealactone, δ-tetralactone; p<0.01). In the same samples there was a reduction of methyl hexanoate (p<0.01) and an increase of methyl octanoate, nonanal, decanal and 1-hexanol (p<0.01).

## DISCUSSION

The daily milk yield was not affected by dietary Zn enrichment. This finding is in agreement to what was previously reported by Pechová et al [27] who supplemented the dairy cows diet with Zn in the chelate form at a dose of 440 mg per animal per day. Moreover, as in our work, authors reported a marked reduction of the SCC in the experimental group, justifying this finding with an increased Zn supply into the mammary gland, with a consequent improvement of the immune function and the reduction of the somatic cells release in milk. In the present study, even the milk composition was not affected by the feeding strategy based on Zn supplementation, and this finding is in agreement with previous reports in dairy cows [28,29] and dairy goats [30], indicating that milk composition was not sensitive to dietary Zn supplementation. This result focusses the attention on the significant difference in the Zn content in milk obtained from the two study groups. Although this finding may seem obvious, it is in contradiction with what was previously reported by Pechová et al [27], who evidenced the inability of dietary Zn supplementation to influence the Zn amount in milk; they discussed such phenomenon advancing the hypothesis of an impaired incidence of rumen acidosis in the herd before the start of the experiment.

According to the results obtained from the analysis of milk, no variations were evidenced in the nutritional composition of cheese samples, both in relation to the feeding strategy and in relation to the ripening time; as expected, the only differences, concerned the increase in dry matter and proteolysis at the end of the ripening period. Particularly interesting is the data concerning the Zn content which is present in higher concentration in the ZG samples, both at C_7_ and at C_120_, according to what was evidenced in milk.

The results of the present study showed the ability of Zn to influence the FA profile of cow milk and related cheese. In this case is evident that the variations highlighted in milk are almost identical to those reported for caciocavallo cheese; in this regard, Nudda et al [31] reported that the FA composition found in cheese reflects the composition observed in milk, suggesting that variations in nutritional quality of milk, obtained as a consequence of the experimental feeding strategy, are thereafter maintained in cheese. Dietary Zn positively affected the oleic acid level in milk and cheese. Since the diet administered to the ZG does not confer an additional source of oleic acid with respect to the CG diet, this result might be mainly related to the desaturation of stearic acid occurring in the mammary gland by stearoyl coenzyme A desaturase (SCD), a finding also supported by the increase of the C14:1/C14:0 ratio, is considered an index of Δ^9^-desaturation in the mammary gland [24]. SCD is an enzyme associated with the endoplasmic reticulum, that catalyzes the Δ^9^-desaturation of satured fatty acyl-CoAs [32]. *SCD* is encoded by the stearoyl coenzyme A desaturase gene [33], whose expression is regulated by the sterol response element binding proteins (SREBP), a transcription factors synthesized as precursor that is attached to the endoplasmic reticulum. The proteolytic cleavage by Site-1 and Site-2 proteases (S1P and S2P, respectively) allows the N-terminal mature and active portion of SREBP to reach the nucleus, inducing the expression of lipogenic factors, including SCD. Of particular interest for our work is the role of S2P, a metalloprotease that needs Zn to perform its catalytic function, justifying, at least in part, the highest concentration of oleic acid in milk and cheese obtained from ZG with respect to CG. Dietary Zn supplementation also induced an increase, both in milk and in caciocavallo cheese, of vaccenic acid and rumenic acid (CLA). The vaccenic acid represents the substrate for the endogenous synthesis of rumenic acid in the mammary gland [34], a pathway which is responsible for the production of about the 60% of rumenic acid released in cow milk. The CLA is reported to perform an important antioxidant activity by mitigating the level of the reactive oxygen species; such function protects bovine mammary epithelial cells from lipoperoxidation, leading to an improvement of the mammary gland functionality [35]. Ruminant products represent for humans the primary dietary source of CLA. The greatest benefits of CLA for human health concern the potential activity of slowing the atherosclerosis development [36], the modulation of the immune system [37] and the enhancement of bone mineralization [38]. Another key to explain the increase in mono and PUFAs could be the effect of Zn in influencing the ruminal biohydrogenation. Engle et al [39] reported the ability of ionophores to inhibit the FA biohydrogenation in rumen, with the consequent decrease in C18:0 and an increase in C18:1 due primarily to an increase in the *trans* C18:1 isomer in ruminal cultures. These alterations in rumen lipid profile were also reflected in milk, although with a smaller magnitude of change [39].

Finally, the increase in MUFA and PUFA at the expense of SFA both in milk and cheese obtained from cows fed the Zn supplementation induced a significant decrease of AI and TI. In light of this, it could be argued that dietary Zn supplementation may increase nutritional value and health functionality of milk and related dairy products.

The determination of TBARS was useful to evaluate the oxidative damage in cheese. Zn has been reported to inhibit free radical lipid peroxidation in biological systems through a mechanism which involves the prevention of OH^•^ and O_2_^−^ production. In particular, Zn has not been proven to directly interact with reactive oxygen species or with carbon-centered free radicals but it has been demonstrated to exert an antioxidant effect by competing with prooxidant metals (i.e., Cu and Fe) for binding sites, thus decreasing their ability to transfer electrons in a particular environment [40]. After only 7 days from the cheese-making, the TBARS values of cheese obtained from the control group were significantly lower with respect to the experimental cheese. At the end of ripening (C_120_), the values increased, as expected, in CG cheese, but they remained almost unchanged in ZG cheese samples suggesting antioxidant protection provided by Zn during the ripening period. A similar behavior was reported by Kahraman and Ustunol with Zn-fortified Cheddar cheese, where TBARS concentrations even decreased over ripening time; this finding was partially justified by introducing the hypothesis of the onset of cross-reactions between the oxidized compounds, which would therefore be underestimated [41].

The evaluation of the aromatic profile in caciocavallo cheese allowed the identification several classes of compounds derived from the lypolitic process; the effect of dietary variation in influencing the aromatic profile of milk and related dairy products has been recently reported [22,26]. The FFA constitute the most represented class of VOCs and are reported to be mainly involved in the determination of cheese flavour, giving origin to cheesy, rancid and sweaty odors; the increased production of such compounds in ZG cheese samples could be probably explained by an increase of lipolysis of the triglycerides by microbial and endogenous milk enzymes, resulting in an augmented release of FFAs [42]. The general increase of carboxylic acids in ripened cheese could be explained by the extent of starter cell autolysis, with the consequent release of peptidases and especially lipases that accelerate the lipolytic event [43]. Over time, several peptidoglycan hydrolases, commonly named autolysins, have been characterized in *Lactococcus lactis*. These enzymes are characterized by an N-terminal domain, resembling a cell-wall-associated domain, a central catalytic domain, and a C-terminal domain containing a binding motif for Zn which is therefore an important cofactor in the mechanisms that lead to the lysis of the bacterial cells [44]. The FFAs contribute to the formation of cheese flavor not only directly, but also giving origin to methyl ketones, aldehydes, lactones and esters, which, in our study, seem to be influenced by dietary Zn supplementation. Ketones may originate from the FFA oxidation to β-ketoacids and subsequent decarboxylation. The biosynthesis of methyl ketones is mainly attributed to mold metabolism, and the accumulation of these compounds is responsible for the typical odors with low perception thresholds, which characterize the surface-mold-ripened and blue-veined cheeses [43]. The reduction of these compounds in the ZG samples at the end of the ripening could derive from the effect of Zn in reducing the enzymatic mechanisms that oversee their synthesis.

Esters are particularly represented in all cheese samples, but they tended to increase in concentration in the exprerimental group during the ripening period. These compounds are characterized by a low odor threshold and are generally associated to the sweet, fruity, and floral notes of surface ripened cheese flavor [45].

The same behavior reported for esters has been observed for lactones which tend to increase in ZG samples during ripening, remaining almost unchanged in CG cheese samples. These compounds are generally produced by a one-step transesterification reaction of hydroxylated FFAs which represent the main precursors. Hydroxylated FFAs are released by lipolytic activities or by heating process, furthermore they can be also be produced from the catabolism of unsaturated FAs by the action of microbial lipoxygenases and hydratases [42]. Probably in the experimental group conditions persisted that favored these enzymatic mechanisms compared to that in CG samples.

Regarding the aldehydes, there was an increase in concen tration of nonanal and decanal both in C_7_ and C_120_ ZG samples. These compounds, in combination with others aldehydes, are responsible for the “green grasslike” aromas, characterized by green, slightly fruity, lemon, and herbal notes [41]. Alcohols are the less represented compounds in cheese samples analyzed in this study. Generally in surface-ripened cheese, alcohols are the VOCs detected in highest numbers, when compared with other classes of compounds. For that reason the synthesis mechanisms and alcohols contribution to the creation of the aromatic profile deserve further evaluation.

## CONCLUSION

The experimented feeding strategy suggests a positive role of Zn in improving the nutritional and nutraceutical properties of milk and corresponding caciocavallo cheese obtained from lactating dairy cows; important indications were obtained that may lead to the improvement of the bovine mammary gland functionality. The main finding of the study concerns the increased amount of vaccenic acid and rumenic acid, indicating that consumption of these products could have positive effects on human health. Besides, the aromatic profile of caciocavallo cheese was also modified by dietary Zn intake. This resulted in an increase in esters and aldehydes, generally responsible for pleasant aromatic notes in aged cheeses. However, the increase in FFAs could contribute to the formation of cheesy, rancid and sweaty odors. For that reason it would be necessary to perform sensorial analysis to verify if these changes may have any effect on consumer acceptability.

## Figures and Tables

**Table 1:** Ingredients and composition of TMR administered to each cow of control and experimental group

Items	Concentrations
Ingredients of TMR	
Corn silage (%)	23.5
First cut, alfalfa hay (%)	5
Corn meal (%)	3.6
Soybean, meal (%)	3.0
Fine bran (%)	3.5
Barley, meal (%)	1.8
CaCO_3_ (%)	0.2
Vitamins and minerals (%)	0.5
kg of dry matter/head/d	22.61
Chemical composition of TMR	
Dry matter (%)	56.66
Crude protein1) (%)	15.33
Ether extract1) (%)	2.96
Ash1) (%)	5.29
Neutral detergent fiber1) (%)	32.65
Acid detergent fiber1) (%)	20.15
Starch1) (%)	26.91
Zinc (mg/head/d)1)	39.1 (+59.6)*

TMR, total mixed ration.

1) On a DM basis.

* In brackets, the amount of zinc added to the diet of the experimental group.

**Table 2:** Milk yield and chemical composition of milk obtained from the control and the experimental group

Items	Diet1)		p value

	CG	ZG	
Animal parameters			
Milk yield (kg/head/d)	40.15±1.56	39.73±1.71	ns
Chemical composition			
Fat (%)	3.66±0.14	3.42±0.16	ns
Protein (%)	3.01±0.12	2.95±0.13	ns
Casein (%)	2.42±0.15	2.45±0.14	ns
Lactose (%)	4.77±0.19	4.80±0.18	ns
Urea (mg/100 mL)	22.78±0.79	23.04±0.72	ns
SCC (×10^3^ cells/mL)	421±55	234±31	**
TBC (UFC/mL, ×10^3^)	55±6	49±7	ns
pH	6.65±0.02	6.67±0.02	ns
Zinc (mg/kg)	3.59±0.23	4.22±0.21	*

SCC, somatic cell count; TBC, total bacterial count; ns, not significant.

1) CG, control group; ZG, zinc group.

* p<0.05,

** p<0.01.

**Table 3:** Chemical composition of cheese obtained from the control and the experimental group, analyzed after 7 (C_7_) and 120 (C_120_) days of ripening

Items	Diet (C_7_)1)		Diet (C_120_)1)	
	
	CG	ZG	CG	ZG
DM (%)	55.11^A^±4.13	53.21^A^±4.32	67.70^B^±4.56	66.21^B^±4.01
Fat (%)2)	39.41±3.02	42.21±3.11	37.70±3.14	37.49±2.92
Protein (%)2)	54.19±3.87	49.97±3.12	49.16±3.37	48.63±3.44
Ash (%)2)	6.86±0.58	7.27±0.62	6.68±0.41	7.09±0.53
SN (% N)2)	0.073^A^±0.008	0.077^A^±0.008	0.12^B^±0.01	0.15^B^±0.02
Zinc (mg/kg)	43.62^a^±1.04	46.50^b^±1.65	41.97^a^±1.55	46.17^b^±2.06

DM, dry matter; SN, soluble nitrogen.

1) CG, control group; ZG, zinc group.

2) Data are expressed on a DM basis.

a,b Means with different superscripts are significantly different by diet (p<0.05).

A,B Means with different superscripts are significantly different by ripening time (p<0.05).

**Table 4:** Fatty acid profile of bulk milk and related cheeses obtained from the control and the experimental group

Items	Milk1)			Cheese1)		
	
	CG	ZG	p value	CG	ZG	p value
C4:0	2.27±0.18	2.56±0.21	ns	2.31±0.20	1.94±0.17	ns
C6:0	2.11±0.17	2.31±0.19	ns	1.86±0.16	1.63±0.14	ns
C8:0	1.36±0.12	1.66±0.14	ns	1.62±0.14	1.33±0.12	ns
C10:0	2.13±0.18	2.02±0.16	ns	2.55±0.22	2.64±0.22	ns
C12:0	4.01±0.29	3.68±0.27	ns	3.81±0.31	3.83±0.33	ns
C14:0	13.32±1.07	11.93±0.98	ns	12.66±0.96	11.91±0.97	ns
C14:1	1.43±0.13	1.78±0.16	ns	1.65±0.15	2.05±0.18	ns
C15:0	1.30±0.11	1.56±0.12	ns	1.57±0.13	1.44±0.12	ns
C16:0	39.27±2.88	34.48±2.66	ns	40.32±3.07	36.17±2.91	ns
C16:1	1.84±0.16	2.31±0.21	ns	1.71±0.15	1.97±0.16	ns
C17:0	0.55±0.05	0.59±0.05	ns	0.68±0.06	0.71±0.07	ns
C18:0	7.41±0.59	6.42±0.61	ns	8.75±0.76	8.19±0.65	ns
C18:1 *trans*11	0.45±0.04	0.81±0.07	**	0.35±0.04	0.76±0.08	**
C18:1 *cis*9	19.47±1.12	23.07±1.89	*	16.72±1.41	21.14±1.94	**
C18:1 *cis*11	0.36±0.03	0.51±0.05	ns	0.45±0.04	0.53±0.05	ns
C18:2	1.74±0.15	2.79±0.22	**	1.97±0.16	2.37±0.19	*
C18:3	0.51±0.05	0.63±0.06	ns	0.52±0.05	0.68±0.07	ns
CLA	0.29±0.03	0.56±0.05	**	0.34±0.04	0.54±0.05	*
C20:4	0.18±0.02	0.23±0.02	ns	0.16±0.02	0.17±0.02	ns
MUFA	23.55±1.92	28.58±2.24	*	20.88±1.31	26.45±2.08	**
PUFA	2.72±0.21	4.21±0.32	**	2.99±0.23	3.76±0.31	*
SFA	73.73±5.86	67.21±4.91	ns	76.13±5.92	69.79±4.77	ns
Atherogenic index	3.68±0.31	2.64±0.24	*	3.97±0.33	2.90±0.25	*
Thrombogenic index	4.16±0.35	3.01±0.27	*	4.59±0.42	3.36±0.30	*
Desaturation index	0.10±0.01	0.13±0.01	*	0.12±0.01	0.15±0.01	*

Data are reported as mean (%)±standard deviation.

ns, not significant; CLA, rumenic acid; MUFA, monounsaturated fatty acid; PUFA, polyunsaturated fatty acid; SFA, saturated fatty acid.

1) CG, control group; ZG, zinc group.

* p<0.05;

** p<0.01.

**Table 5:** Volatile compounds (VOCs) detected in C_7_ and C_120_ cheese samples obtained from control and experimental group

Items	VOC	C_7_1)			C_120_1)		
	
		CG	ZG	p value	CG	ZG	p value
Methyl ketones	2-pentanone	0.46±0.05	1.16±0.09	**	0.34±0.04	0.09±0.01	**
	2-heptanone	18.73±1.22	21.80±1.83	ns	44.66±3.56	7.05±0.59	**
	2-octanone	0.56±0.06	0.49±0.05	ns	2.73±0.23	0.32±0.03	**
	2-nonan-2-one	nd	nd	ns	2.33±0.19	0.29±0.03	**
	2-nonanone	6.66±0.54	7.60±0.63	ns	29.46±1.91	10.89±0.87	**
	2-decanone	nd	nd	ns	nd	nd	ns
	2-undecanone	1.66±0.14	2.37±0.20	*	nd	nd	ns
	2-tridecanone	0.39±0.04	0.45±0.05	ns	nd	nd	ns
Methyl esters	methyl butanoate	0.23±0.03	0.54±0.05	**	nd	nd	ns
	methyl hexanoate	0.29±0.03	0.42±0.04	*	0.33±0.03	0.22±0.02	**
	methyl octanoate	0.18±0.02	0.18±0.01	ns	0.04±0.00	0.12±0.01	**
Ethyl esters	ethyl butanoate	2.18±0.17	1.34±0.12	**	2.77±0.21	8.47±0.76	**
	ethyl hexanoate	1.25±0.10	0.69±0.06	**	8.58±0.69	29.09±2.11	**
	ethyl octanoate	0.65±0.07	0.47±0.05	*	1.89±0.16	15.22±1.12	**
	ethyl nonanoate	nd	nd	ns	0.05±0.00	0.44±0.04	**
	ethyl decanoate	0.37±0.04	0.41±0.04	ns	0.79±0.08	10.11±0.89	**
	ethyl dodecanoate	0.10±0.01	0.08±0.01	ns	0.12±0.01	2.37±0.18	**
	ethyl tetradecanoate	nd	nd	ns	nd	0.51±0.05	**
	ethyl hexadecanoate	nd	nd	ns	nd	0.15±0.02	**
Lactones	cis-γ-dodec-6-enolactone	nd	nd	ns	0.09±0.01	0.29±0.03	**
	γ-nonalactone	nd	nd	ns	0.08±0.01	0.16±0.02	**
	γ-dodecalactone	0.67±0.07	0.83±0.08	ns	0.30±0.03	0.99±0.01	**
	δ-octalactone	4.91±0.36	2.56±0.22	**	nd	nd	ns
	δ-nonalactone	nd	nd	ns	0.65±0.06	0.73±0.07	ns
	δ-decalactone	14.54±1.03	16.26±1.44	ns	2.33±0.19	5.53±0.37	**
	δ-dodecalactone	2.37±0.19	2.72±0.25	ns	0.51±0.05	1.58±0.14	**
	δ-tetralactone	0.19±0.02	0.21±0.02	ns	0.04±0.00	0.15±0.01	**
Aldehydes	hexanal	33.86±2.87	27.20±2.24	*	nd	nd	ns
	heptanal	2.78±0.24	3.17±0.26	ns	0.55±0.06	0.63±0.06	ns
	octanal	0.59±0.06	0.72±0.07	ns	0.12±0.01	0.14±0.01	ns
	nonanal	2.22±0.18	3.71±0.31	**	0.44±0.04	0.74±0.07	**
	decanal	0.54±0.05	1.12±0.10	**	0.11±0.01	0.22±0.02	**
Alcohols	1-hexanol	2.74±0.23	2.54±0.21	ns	0.67±0.07	3.49±0.24	**
	1-octanl	0.90±0.09	0.96±0.09	ns	nd	nd	ns

VOCs, volatile compounds; ns, not significant; nd, not detectable.

1) CG, control group; ZG, zinc group.

* p<0.05;

** p<0.01.
